# Psychometric Properties of the SOC-13 Scale in Colombian Adults

**DOI:** 10.3390/ijerph182413017

**Published:** 2021-12-10

**Authors:** Ana Cristina Mafla, Mauricio Herrera-López, Karen España-Fuelagan, Iván Ramírez-Solarte, Carmen Gallardo Pino, Falk Schwendicke

**Affiliations:** 1 School of Dentistry, Universidad Cooperativa de Colombia, Pasto 520004, Colombia; ana.mafla@campusucc.edu.co (A.C.M.); karen.espanaf@campusucc.edu.co (K.E.-F.); ivan.ramirezs@campusucc.edu.co (I.R.-S.); 2 Escuela Internacional de Doctorado, Universidad Rey Juan Carlos, 28922 Madrid, Spain; 3 Department of Psychology, Universidad de Nariño, Pasto 520004, Colombia; mherrera@udenar.edu.co; 4 Departamento de Especialidades Médicas y Salud Pública, Facultad Ciencias de la Salud, Universidad Rey Juan Carlos, 28922 Madrid, Spain; carmen.gallardo@urjc.es; 5 Department of Oral Diagnostics, Digital Health and Health Services Research, Charité—Universitätsmedizin Berlin, 14197 Berlin, Germany

**Keywords:** sense of coherence, SOC-13, cross-validation, factor analysis, multi-group analysis

## Abstract

The aim of this study was to determine the psychometric properties of the Spanish version of the SOC-13 in Colombian adults. The SOC-13 questionnaire was administered to 489 individuals aged ≥18 years who were in lockdown from March to July 2020 in Nariño County, Colombia. Psychometric properties of the scale were examined using a cross-validation method via exploratory factor analysis (EFA) and confirmatory factor analysis (CFA). Additionally, configural and metric invariance were tested. To determine the internal consistency of the questionnaire, McDonald’s omega (ω), Cronbach’s alpha (α), and composite reliability (CR) coefficients were estimated. The EFA determined that a three-factor structure best fit the data (comprehensibility, manageability, and meaningfulness) and CFA confirmed this three-factor model structure showing a good fit (*χ*^2^_S-B_ = 188.530, *χ*^2^_S-B_/(62) = 3.615, *p* = 0.001; *NNFI* = 0.959; *CFI* = 0.968; *RMSEA* = 0.052 (90% CI [0.041–0.063]) and *SRMR* = 0.052).The invariance analysis indicated the same underlying theoretical structure between genders. Additionally, (ω), (α), and (CR) coefficients confirmed a high internal consistency of the instrument. The SOC-13 scale, reflecting comprehensibility, manageability, and meaningfulness, is a reliable and valid tool for assessing the sense of coherence in Colombian populations.

## 1. Introduction

The sense of coherence is defined as the ability to perceive the meaning of the world in a clear and structured way and as the comprehension of the relationship between actions and consequences. It is a valuable resource to deal with stressors [1]; this ability has been related to quality of life and can be considered as a predictor of health. The latter is grounded in individuals’ capacities to assess and understand the situation of their health (comprehensibility), allowing them to find a meaning (meaningfulness) to move in a health-promoting direction (manageability) [2,3].

The comprehensibility component is defined as the degree to which internal and external stimuli are coherent, structured, and comprehensible for people [4]. The manageability element is related to peoples’ perception that they have enough internal (e.g., cognitive, emotional and behavioral strategies) as well as external resources (e.g., social support, social fairness, relationships, outdoor life, culture to cope with difficulties and maintain good health) to meet their demands [5]. Finally, meaningfulness is a dimension that refers to the extent to which individuals believe that the demands are worthy of investment and engagement [6]. According to Antonovsky [2], a high degree of sense of coherence allows individuals to move from a condition of illness to one of health; they conceive new activities as comprehensible and manageable. A strong sense of coherence boosts resilience and increments individual well-being.

A higher level of sense of coherence has been linked to different phases of diabetes, from risk control until reduction of glycated hemoglobin values and complications [7]. This ability has also been linked to resolving addictions [8] and depression [9], improving the quality of life in patients with head and neck cancer [10] or dealing with mental health-related quality of life problems because of sickness [11]. Some studies in dentistry have associated a high sense of coherence with lower caries experience in adolescents [12], less dental pain in women [13] or generally better self-assessed oral health [14].

To evaluate individuals’ sense of coherence, Antonovsky [1], developed a 29-item questionnaire which consisted of 11 items that measured comprehensibility, 10 manageability and eight meaningfulness. Later, this author proposed a reduced sense-of-coherence scale version of 13 items (SOC-13). The scale uses a Likert-type rating scale that ranged from 1 (never) to 7 (always) [15]. Since the scale is ordinal and assumes that the strength/intensity of this ability is linear, higher sum scores indicate a higher sense of coherence.

The SOC-13 questionnaire has shown good psychometric properties. While there was evidence that the scale, after some adaption, loads onto one factor [16], a range of studies found SOC to be multidimensional instead. Validation studies in Indian college students [17], in Slovenian patients with multiple sclerosis [18] and in older adults confirmed a three-factor structure [19]. However, in some studies one or two items have been removed because the one- or three-factor structure fits better with 11 or 12 items.

The SOC-13 scale has been proposed as a reliable and valid screening instrument for sense of coherence with one latent factor. Nevertheless, studies have found a model that supports a three-dimensional structure according to Antonosvky’s theory [2]. Since in Colombia there were few cultural validation studies of the SOC-13 questionnaire and we observed some limitations of this instrument in different contexts, a validation of this instrument was needed. Considering all this, the main goal of the present study was to examine the psychometric properties of the Spanish version of the SOC-13 scale (Annex 1) in Colombian adults.

## 2. Materials and Methods

### 2.1. Study, Participants and Settings

The present study is a cross-sectional survey of an adult population living in Nariño, Colombia. The sample comprised of friends and relatives of five-year dental students from Universidad Cooperativa de Colombia. This dental school is one of four founded by the university and is located in southern Colombia.

The data were collected from May to July 2020. A total of 552 individuals were invited and 489 (88.6%) agreed to participate. Voluntary adults ≥18 years-old, who were or not in lockdown due to COVID-19 and willing to cooperate with relevant aspects of the study, were included. Individuals who did not answer the invitation after two weeks were excluded.

### 2.2. Instrument

#### Sense of Coherence Scale (SOC-13)

The Sense of Coherence questionnaire was developed by Antonovsky [20]. The original scale was the Orientation to Life Questionnaire (OLQ), consisting of 29 items that measure Comprehensibility, Manageability, and Meaningfulness with Likert response alternatives from 1 point to 7 points, where 1 (never) and 7 (always) indicate extreme feelings about one’s life experiences. A shorter version of 13 questions (SOC-13) of the original form was developed by the author [15]. The SOC-13 scale consists of some items in the subscales as follows: Comprehensibility (5 items: 2, 6, 8, 9 and 11), Manageability (4 items: 3, 5, 10 and 13), and Meaningfulness (4 items: 1, 4, 7 and 12). The final score of the scale for each participant score includes a reversed score of questions 1, 2, 3, 7 and 10 (where score 7 = 1, 6 = 2, 5 = 3, 4 = 4, 3 = 5, 2 = 6 and 1 = 7). The score ranges from 13 to 91 points, and higher total scores indicate a stronger sense of coherence. We used a Spanish version of the SOC–13 questionnaire which was translated and validated in this language [21], reporting a Chronbach’s (α) coefficient of 0.80. Additionally, we ensured that all items of this instrument had semantic and technical equivalence to the original version [22]. Firstly, in order to determine the semantic equivalence of each item, the researchers counted the number of words of the instrument. A total of 182 words were overlapping (pairs of words of Spanish from Spain and Spanish from Colombia); among them, 172 (94.5%) had the same equivalence and 10 words (5.5%) did not. In item 10, the word “perdedores” (“losers”) was used instead of “desgraciados” (“despicable”) due to different connotations. The meaning of “perdedor” (“loser”) in a Colombian context is close to “a person who is incompetent or unable to succeed”; however, “desgraciado” (“despicable”) is a pejorative word that means “a person who deserves to be despised”. A linguistic assessment was performed for 9 words of the questionnaire as well. The word “Usted” (“You”) was utilized instead of the abbreviation “Vd.”; even though both mean the same, the latter is less recognized not only in Colombia but generally in Latin America. Secondly, a technical equivalence assessment was carried out. The personal pronoun “le” used in the items was appropriate because, according to the socio-cultural context, a formal personal pronoun is employed in questionnaires. Moreover, as a part of this process, the length and complexity of the sentences were evaluated, and researchers observed that the items in general were short and simple. The longest item had 26 words (item 10) and the shortest had 4 words (item 4). The questionnaire was pre-tested in 5% of the sample (25 participants) to evaluate whether the questions were clear. Three dichotomous questions (yes/no) were asked for each item, such as 1) “I understand the question”, 2) “I understand but a change needs to be made in this question” (please explain) and 3) “I do not understand this question” (please explain). No changes were made after this process. We obtained permission to use this version through the Society for Theory and Research on Salutogenesis (STARS).

The format also included demographic characteristics of the participants such as age (measured in years and classified in three groups (18–24 years), (25–34 years) (≥35 years)); gender (coded as male and female according to the World Health Organization definition) [23]; socioeconomic status (SES), classified according to criteria based on housing quality indicators set by the Colombian government [24] (coded as low, middle and high); permanent residency (coded as Pasto (capital) and other place); education (coded as primary, high school and university) and health insurance (coded as subsidized and non-subsidized).

### 2.3. Procedures

Participants were recruited online (WhatsApp, Mountain View, CA, USA). Prior to sending the survey, participants were contacted in order to obtain their verbal consent, and to explain to them the study procedures and purpose. The set time for answering the questionnaire was between 5 and 12 min. The participants were encouraged to ask the researchers if they had any doubt about the different items of the scale any time.

### 2.4. Statistical Approach

Descriptive analyses were calculated to determine the distribution of demographic variables in the entire population and describe the SOC-13 items scale measures such as skew, kurtosis, and inter-item correlations. We verified the multivariate normality assumption of the data through Mardia’s coefficient.

To validate the questionnaire, the sample was randomly split into equivalent subsets to conduct factor analyses with a cross-validation analysis. The main purpose of this analysis was to explore the item distribution and confirms the theoretical model of the measurement [25,26]. In the first subsample, an Exploratory Factor Analysis (EFA) was conducted, using the principal axis method with oblique (direct oblimin) rotation, in order to detect a cluster structure that may be present among items. Sampling adequacy indices such as Kaiser–Meyer–Olkin (KMO), Bartlett’s sphericity, communalities values, item saturations and factor loadings (obtained from the free items distribution according to the matrix of configuration) were estimated in this analysis. The EFA was performed using the Factor 9.2 statistical package [27].

In the second subsample, a Confirmatory Factor Analysis (CFA) was conducted to test the fit of the factor structures using a Robust Least Square (RLS) method. Since we were working with ordinal (categorical) variables and the data were not multivariate normally distributed, polychoric correlations were calculated [28,29]. The following indices were estimated to evaluate model fit: Satorra–Bentler Chi^2^ (*χ*^2^_S-B_) [30], Chi^2^_S-B_/degrees of freedom (*χ*^2^_S-B_/df) (≤3), the Non-normed Fit Index (*NNFI*) (≥0.95), Comparative Fit Index (*CFI*) (≥0.95), the Root Means Square Error of Approximation (*RMSEA* ≤ 0.08), and the Standardized Root Mean Residual (*SRMR*) (≤0.08) [31]. These analyses were performed with the EQS 6.2 statistical package [32].

Additionally, configural and metric invariance were assessed in different genders. Metric invariance was tested with a constrained or restricted method (Model I). This analysis compared the fit indices of the restricted (Model I) to those from the non-restricted model. Parameters for invariance hypothesis rejection were delta (Δ) of fit measures (Δ*NNFI*, Δ*CFI*, Δ*RMSEA* and Δ*SRMR*) (≤0.01) [33]. Finally, a chi square difference test (Δ*χ*^2^_S-B_) was used; a non-significant value of this measure indicated invariance in the model [34,35].

McDonald’s omega (ω) coefficient [36] was used to assess the internal consistency, a suggested measure to approach ordinal (categorical) and not multivariate normally distributed data. This coefficient was calculated by using the Factor version 9.2 statistical package [27]. To complement this analysis, Chronbach’s (α) and composite reliability (CR) coefficients were estimated to evaluate the factorial structure. The coefficients’ cut-off point for inferring an adequate internal consistency was set at ≥0.70. The level of significance was set at *p* < 0.05.

## 3. Results

### 3.1. Sociodemographic Characteristics

The age of participants ranged from 18 to 91 years (*mean* = 36.01; *standard deviation, SD* = 15.36 years). A total of 142 (29%) participants were between 18–24 years-old, 148 (30.3%) between 25–34 years-old and 199 (40.7%) were ≥35 years-old. The sample consisted of 263 (57.8%) males and 226 (46.2%) females. An amount of 247 (50.5%) belonged to a low socioeconomic status (SES), 189 (38.7%) to middle SES and 53 (10.8%) to high SES. Regarding permanent residency, 224 (45.8%) lived in Pasto city (capital) and 265 (54.2%) in other places. A total of 288 (58.9%) participants were single and 201 (41.1%) were married. Regarding education, 45 (9.2%) had completed primary school, 150 (30.7%) high school, and 294 (60.1%) university studies. A total of 207 (42.3%) individuals reported having public health insurance and 282 (57.7%) private health insurance.

### 3.2. Psychometric Properties of the SOC-13 Scale

Descriptive analyses for the scale and items are shown in Table 1. The mean of the SOC-13 was 63.56, *SD* = 11.64. Mardia’s analysis demonstrated a skewness of 12.71, *p* < 0.001 and kurtosis of 252.17, *p* < 0.001 indicating that the data did not have a multivariate normal distribution.

To validate the construct of the Colombian version scale, an exploratory factor analysis (EFA) was initially performed, since it was important to determine if the items were distributed according to a single factor, as was proposed in the original study. The results indicated a sample adequacy Kaiser–Meyer–Olkin (KMO) test result of 0.79 and a significant Bartlett sphericity test (*χ*^2^ = 1316.20; df = 78; *p* ≤ 0.001). The communalities were adequate, ranging between 0.310 (item 7) and 0.661 (item 2) (Table 2).

**Figure 1:**
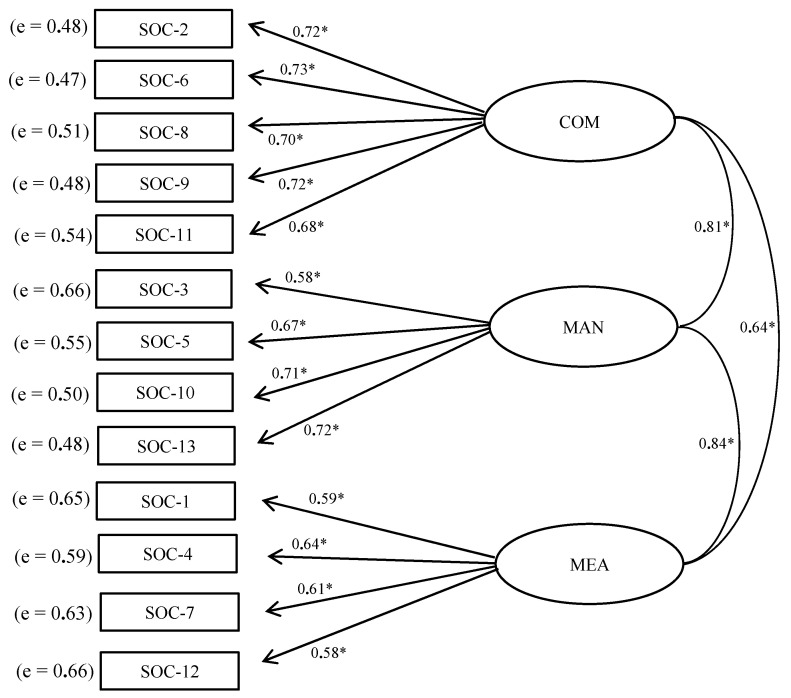
Path diagram of Confirmatory Factor Analysis for the SOC-13 scale Colombian version. COM = comprehensibility, MAN = manageability, MEA = meaningfulness. (* *p* < 0.05).

After obtaining results from the EFA, the three-factorial structure was examined through a CFA. The goodness of fit indices of the SOC-13 questionnaire confirmed optimal adequateness (*χ*^2^_S-B_ = 188.530, *χ*^2^_S-B_/(62) = 3.615, *p* = 0.001; *NNFI* = 0.959; *CFI* = 0.968; *RMSEA* = 0.052 (90% CI [0.041–0.063]) and *SRMR* = 0.052). Moreover, *lambda* (λ) values, determination coefficients (R^2^) and measurement errors (*e*) were also adequate (Figure 1).

The multi-group analyses showed non-significant differences by gender according to invariance measurements (Supplemental Table S1). Finally, the coefficients indicated high internal consistency for the total scale (ω = 0.78; α = 0.76; CR = 0.91).

## 4. Discussion

While the SOC-13 questionnaire has been validated in Spanish (mainland Spain) before [21], we aimed to determine the psychometric properties of the instrument in another Spanish-speaking population, mainly as we were interested in assessing if the structure of the scale presented similar characteristics (e.g., 4-, 3- or 1-factor loading) [37,38], but also because language usage differs in some aspects between different Spanish speaking areas (e.g., Spain versus Latin American countries).

This study was designed to determine the psychometric properties of the questionnaire in a Colombian population. The Spanish-language version of the scale proved to be valid and reliable. In the descriptive analysis of the scale, items 1 and 7 showed lower mean scores; those represent higher values because they are reverse items. These items show that this sample cares about what goes on around them and the things they undertake every day are a source of satisfaction. The participants also had higher mean scores in item 4, denoting that until now their life has had very clear goals or purposes.

A cross-validation analysis was used to assess the stability of the statistical model. The results of the EFA determined a three-factor structure with adequate communalities and factor saturations. A similar result in a group of nursing students from Spain was observed [39]. However, in the last study, the factors did not have the same items proposed by Antonovsky [4] for dimensions. CFA based on goodness-of-fit indices confirmed this three-factorial structure, providing evidence for construct validity. For this reason, according to psychometric properties and theoretical reasons, a three-factor structure of the SOC-13 scale should be used to assess the sense of coherence in Colombian populations. This structure was different to that reported by Virués et al. [21], where their analysis showed a two-factor solution in older people from Spain. Additionally, the invariance analysis indicated that the measurement of sense of coherence with the Colombian version of the SOC-13 has the same underlying theoretical structure between genders.

The SOC-13 questionnaire has been found to come with high internal consistency (Cronbach’s α from 0.70 in previous studies) [38], showing a good degree of interrelationship or homogeneity among the items of the scale. We confirm this and further support the notion of high consistency via McDonald’s omega and CR coefficients.

### Limitations

This study presented some limitations that can have implications for further research as well. Firstly, we did not perform a transcultural adaptation process for the SOC-13 scale into Spanish from Colombia, as we found some differences in this language translation. Secondly, we did not include other psychometric properties such as test–retest reliability of the scale to ensure that these properties are stable over time. Thirdly, even though our sample was appropriated for our study [40], it was not randomly distributed; thus, the findings might not be generalized to the population. Fourthly, data collection was based on a self-report questionnaire; for this reason, a social desirability bias might exist despite the fact that it had been completely anonymous. Finally, psychometric analysis, as well as valid scales equivalence across cultures, should be a continuing process that requires regular assessment in different contexts.

## 5. Conclusions

The SOC-13 is a questionnaire that can be used with three factors, such as comprehensibility, manageability, and meaningfulness, being a credible tool to measure sense of coherence in Colombian populations. The SOC-13 scale may be appropriate and used in clinical research, as well as clinical practice, but further research is needed to evaluate its usefulness in other populations and clinically in patients with different diseases.

## Figures and Tables

**Table 1:** Descriptive analysis of the SOC-13 items (frequencies, means, standard deviations, skewness and kurtosis).

Item	M	*SD*	Skewness	Kurtosis	1 *n*/%	2 *n*/%	3 *n*/%	4 *n*/%	5 *n*/%	6 *n*/%	7 *n*/%
**SOC-1.** Do you have the feeling that you really don’t care about what is going on around you?	3.32	2.04	0.38	−1.16	137/28	77/15.7	58/11.9	60/12.3	70/14.3	41/8.4	46/9.4
**SOC-2.** Has it happened in the past that you were surprised by the behavior of people whom you thought you knew well?	4.37	1.73	−0.16	−0.79	33/6.7	37/7.6	89/18.2	87/17.8	113/23.	58/11.9	72/14.7
**SOC-3.** Has it happened that people whom you counted on disappointed you?	4.08	1.84	0.04	−1.0	43/8.8	72/14.7	77/15.7	93/19	89/18.2	46/9.4	69/14.1
**SOC-4.** Until now your life has had:	5.70	1.45	−1.20	0.91	7/1.4	14/2.9	25/5.1	46/9.4	73/14.9	138/28.2	186/38
**SOC-5.** Do you have the feeling that you are being treated unfairly?	5.07	1.73	−0.75	−0.35	23/4.7	29/5.9	43/8.8	60/12.3	95/19.4	118/24.1	121/24.7
**SOC-6.** Do you have the feeling that you are in an unfamiliar situation and don’t know what to do?	5.19	1.76	−0.82	−0.35	20/4.1	33/6.7	39/8	56/11.5	77/15.7	121/24.7	143/29.2
**SOC-7.** Doing the things you do every day is…	3.32	1.94	0.40	−1.11	112/22.9	103/21.1	62/12.7	66/13.5	55/11.2	58/11.9	33/6.7
**SOC-8.** Do you have very mixed-up feelings and ideas?	5.25	1.67	−0.78	−0.35	12/2.5	31/6.3	40/8.2	65/13.3	73/14.9	127/26	141/28.8
**SOC-9.** Does it happen that you experience feelings that you would rather not have to endure?	4.98	1.78	−0.59	−0.71	21/4.3	36/7.4	56/11.5	62/12.7	82/16.8	107/21.9	125/25.6
**SOC-10.** Many people, even those with a strong character, sometimes feel like losers in certain situations. How often have you felt this way in the past?	3.04	1.58	0.64	−0.22	84/17.2	131/26.8	96/19.6	95/19.4	42/8.6	23/4.7	18/3.7
**SOC-11.** When certain events occurred, have you generally found that:	4.72	1.77	−0.43	−0.78	26/5.3	41/8.4	55/11.2	84/17.2	94/19.2	92/18.8	97/19.8
**SOC-12.** How often do you have the feeling that there is little meaning in the things you do in your daily life?	5.56	1.58	−1.14	0.42	8/1.6	30/6.1	28/5.7	36/7.4	72/14.7	143/29.2	172/35.2
**SOC-13.** How often do you have feelings that you are not sure you can control?	5.20	1.76	−0.85	−0.39	17/3.5	41/8.4	41/8.4	39/8	78/16	134/27.4	139/28.4

***Item responses.* SOC-1**: 1 = “Very seldom or never”—7 = “Very often”. **SOC-2** and **SOC-3**: 1 = “Never happened”—7 = “Always happened”. **SOC-4**: 1 = “No clear goals”—7 = “Very clear goals and purpose”. **SOC-5**, **SOC-6**, **SOC-8**, **SOC-9**, **SOC-12** and **SOC-13**: 1 = “Very often”—7 = “Very seldom or never”. **SOC-7**: 1 = “A source of deep pleasure and satisfaction”—7 = “A source of pain and boredom”. **SOC-10**: 1 = “Never”—7 = “Very often”. **SOC-11**: 1 = “You overestimated or underestimated their importance”—7 = “You saw the things in the right proportion”. *Note: n*/% = frequency/percentage.

**Table 2:** Exploratory Factor Analysis using a principal axis analysis with oblique (direct oblimin) rotation of the SOC-13 scale (Colombian version).

Item	Factor 1	Factor 2	Factor 3	h^2^ Communalities
SOC-2	0.804			0.661
SOC-6	0.675			0.533
SOC-8	0.711			0.437
SOC-9	0.606			0.581
SOC-11	0.411			0.566
SOC-3		0.797		0.630
SOC-5		0.518		0.555
SOC-10		0.460		0.591
SOC-13		0.661		0.632
SOC-1			0.341	0.479
SOC-4			0.700	0.562
SOC-7			0.422	0.310
SOC-12			0.632	0.588

## Data Availability

The data presented in this study are available on reasonable request from the corresponding author.

## Supplementary Materials

The following are available online at https://www.mdpi.com/article/10.3390/ijerph182413017/s1, Supplemental Table S1. Configural and metric invariance of the SOC-13 questionnaire by gender. Annex 1. Spanish version of the SOC-13 scale (Colombian version).
