# Supplementary material for: Psychometric Properties of the SOC-13 Scale in Colombian Adults
Source: Int J Environ Res Public Health. 2021 Dec 10;18(24):13017. doi: 10.3390/ijerph182413017 (PMC8700993; doi:10.3390/ijerph182413017)
Supplement: Supplementary file 1 [file ijerph-18-13017-s001.zip › ijerph-1405803-Supplementary.pdf]

Supplemental Table S1. Configural and metric invariance of the SOC-13 questionnaire by gender

	Model	χ^2_{S-B}	*df*	*P*	NNFI	CFI	RMSEA	SRMR	$\Delta\chi^2_{S-B}$	ΔNNFI	ΔCFI	ΔRMSEA	ΔSRMR
**Sex**	Males	202.610	62	0.000	0.958	0.966	0.058 [0.054, 0.064]	0.061					
	Females	163.715	62	0.000	0.967	0.972	0.051 [0.046, 0.065]	0.060					
**Multi-group**	Non-restricted	188.530	62	0.001	0.959	0.968	0.052 [0.045, 0.060]	0.052	--	--	--	--	--
	Model I restricted	125.921	51	0.000	0.947	0.962	0.048 [0.040, 0.058]	0.061	62.609[a]	0.001	0.000	0.000	0.010

Note: [a]= non-significant

Annexe 1

Spanish version of the SOC-13 scale (Colombian version)

A continuación, hay una serie de preguntas sobre varios aspectos de la vida. Cada pregunta tiene 7 respuestas posibles. Marque el número que mejor exprese su pensamiento. Por favor, seleccione sólo una respuesta para cada pregunta.

**1**. ¿Tiene la impresión de que a Usted no le importan de verdad las cosas que pasan a su alrededor?						
**Rara vez o nunca** Mínimo valor						**Muy a menudo** Máximo valor
1	2	3	4	5	6	7
**2**. ¿Le ha sorprendido alguna vez lo que han hecho personas que Usted creía que conocía bien?						
**Nunca me ha pasado** Mínimo valor						**Siempre me pasa** Máximo valor
1	2	3	4	5	6	7
**3**. ¿Le ha pasado alguna vez que personas con las que contaba le hayan defraudado?						
**Nunca me ha pasado** Mínimo valor						**Siempre me pasa** Máximo valor
1	2	3	4	5	6	7
**4**. Hasta ahora su vida…						
**No ha tenido en absoluto objetivos o metas claras** Mínimo valor						**Ha tenido objetivos y metas claras** Máximo valor
1	2	3	4	5	6	7
**5**. ¿Tiene Usted la impresión de que le están tratando de forma injusta?						
**Muy a menudo** Mínimo valor						**Rara vez o nunca** Máximo valor
1	2	3	4	5	6	7
**6**. ¿Tiene Usted la impresión de estar en una situación a la que no está acostumbrado/a y no sabe qué hacer?						
**Muy a menudo** Mínimo valor						**Rara vez o nunca** Máximo valor
1	2	3	4	5	6	7

**7**. Hacer las cosas que Usted hace todos los días…						
**Me produce una gran alegría y satisfacción** Mínimo valor						**Me produce dolor y aburrimiento** Máximo valor
1	2	3	4	5	6	7
**8**. ¿Tiene Usted sentimientos o ideas muy confusas?						
**Muy a menudo** Mínimo valor						**Rara vez o nunca** Máximo valor
1	2	3	4	5	6	7
**9**. ¿Le pasa que tiene sentimientos dentro de Usted que preferiría no tener?						
**Muy a menudo** Mínimo valor						**Rara vez o nunca** Máximo valor
1	2	3	4	5	6	7
**10**. Muchas personas, hasta los que tienen un carácter fuerte, se sienten a veces como unos perdedores. ¿Cuántas veces se ha sentido Usted así en su vida?						
**Nunca** Mínimo valor						**Muy a menudo** Mínimo valor
1	2	3	4	5	6	7
**11**. Cuando algo le ha pasado, al final ha visto Usted que…						
**Le dio más importancia o menos importancia de la que en verdad tenía** Mínimo valor						**Dio a las cosas la importancia justa** Máximo valor
1	2	3	4	5	6	7
**12**. ¿Cuántas veces tiene la impresión de que las cosas que hace todos los días significan muy poco o tienen poca importancia?						
**Muy a menudo** Mínimo valor						**Rara vez o nunca** Máximo valor
1	2	3	4	5	6	7
**13**. ¿Cuántas veces tiene la impresión de no estar seguro/a de poder controlarse?						
**Muy a menudo** Mínimo valor						**Rara vez o nunca** Máximo valor
1	2	3	4	5	6	7

Puntaje: Los ítems 1, 2, 3, 7 y 10 se contabilizan de manera inversa. Es decir 1=7, 2=6, 3=5, 4=4, 5=3, 6=2 y 7=1. Los demás se suman como aparecen marcados.

Copyright. Permission to use the scale is granted by Society for Theory and Research on Salutogenesis (STARS). https://www.stars-society.org/
